# *Rosa26*-targeted sheep gene knock-in via CRISPR-Cas9 system

**DOI:** 10.1038/srep24360

**Published:** 2016-04-11

**Authors:** Mingming Wu, Caihong Wei, Zhengxing Lian, Ruizao Liu, Caiye Zhu, Huihua Wang, Jiaxue Cao, Yuelei Shen, Fuping Zhao, Li Zhang, Zhu Mu, Yayu Wang, Xiaogang Wang, Lixin Du, Chuduan Wang

**Affiliations:** 1 College of animal science and technology, China Agricultural University, Beijing 100193, China; 2 Institute of Animal Sciences, Chinese Academy of Agricultural Sciences, Beijing 100193, China; 3 Beijing Biocytogen Co., Ltd, Beijing 100176, China; 4 College of life science and technology, Jinan university, Guangzhou 510632, China

## Abstract

Recent advances in our ability to design DNA binding factors with specificity for desired sequences have resulted in a revolution in genetic engineering, enabling directed changes to the genome to be made relatively easily. Technologies that facilitate specific and precise genome editing, such as knock-in, are critical for determining the functions of genes and for understanding fundamental biological processes. The CRISPR/Cas9 system has recently emerged as a powerful tool for functional genomic studies in mammals. Rosa26 gene can encode a non-essential nuclear RNA in almost all organizations, and become a hot point of exogenous gene insertion. Here, we describe efficient, precise CRISPR/Cas9-mediated Integration using a donor vector with tGFP sequence targeted in the sheep genomic Rosa26 locus. We succeeded in integrating with high efficiency an exogenous tGFP (turboGFP) gene into targeted genes in frame. Due to its simplicity, design flexibility, and high efficiency, we propose that CRISPR/Cas9-mediated knock-in will become a standard method for the generation transgenic sheep.

Sheep is an important economic animals and production trait has become the main direction of sheep breeding. Transgenic animals with reporter gene expression in specific tissues or cell types are valuable experimental tools. Recently, programmable endonucleases, such as Zinc Finger Nucleases (ZFNs), Transcription activator-like effector nucleases (TALENs), and especially the CRISPR, the recently developed clustered regularly interspaced short palindromic repeats (CRISPR)/CRISPR-associated (Cas) 9 system, were shown to be a kind of revolutionary technologies for targeted genome editing^1^. CRISPR loci contain an array of short palindromic repeats separated by proto spacer DNA snippets of about 20 bp that have been acquired from such invading agents. Compared to ZFNs and TALENs, CRISPR/Cas9-mediated genome engineering is easy to handle, highly specific, efficient, and multiplexable^2^. Compared with protein-based genome editing tools with customizable DNA binding specificities, such as ZFNs and TALENS, the newer CRISPR/Cas9 platform is based on simple base-pairing between an engineered RNA and the targeted genomic site, which enables rapid design, ease of use, and low costs^3^. The Cas9 endonuclease is ushered to the specific site of interest by a single guide RNA (sgRNA), an engineered fusion molecule of the targeting CRISPR RNA (crRNA) and the trans-activating crRNA, to generate double-stranded DNA breaks (DSDBs) at the target site^4^. The CRISPR/Cas9 system has been demonstrated to be easy to handle, highly specific, efficient, and multiplexable, making it a suitable approach to the engineering of eukaryotic genomes^5^. This system has been successfully used to target genomic loci in several species, including mice, rats, zebrafish, and pigs^6^,^7^,^8^,^9^,^10^.

However, so far, there have been no reports of the use of CRISPR/Cas9-mediated knock-in DNA cassettes in the sheep genome. ROSA26 is a safe area, exogenous genes that decide a dot inside this site will not affect the expression of other genes. *Rosa26* is ubiquitously expressed in embryonic and adult tissues^11^. *Rosa26* was first identified and targeted in mouse embryonic stem cells (ESCs) in the 1990s and then in human ESCs in 2007^10^. Efficient integration of transgenes at preselected chromosomal locations was achieved in mammalian cells by recombinase-mediated-cassette-exchange (RMCE), a novel procedure that makes use of the CRE recombinase together with Lox sites bearing different spacer regions. Here, the sheep Rosa26 (sRosa26) locus was characterized and its locus tagged with a knock-in reporter gene using a CRISPR/Cas9-mediated method. Using this approach, transgenic sheep stably over-expressing a gene of interest were also created through RMCE^12^.

The most preferred integration site used for transgene insertionis the Rosa26 locus in mice. As in mice, the human and rat Rosa26 loci were identified and successfully targeted using traditional HR^13^. There have reported that knock-in of a long DNA fragment via homology-independent DNA repair can be achieved in zebrafish using the CRISPR/Cas9 system. In this method, co-injection of a donor plasmid, short guide RNAs (sgRNAs) and Cas9 mRNA leads to concurrent digestion of the genomic DNA and the donor plasmid, resulting in the incorporation of the donor plasmid into the genome^14^,^15^. In some cases, fluorescent proteins such as GFP have been used for the characterisation and quantification of homologous recombination events as well as for endogenous gene-tagging and determination of subcellular localisation of expressed proteins^16^.

Here, we have modified this method, and succeeded in generating knock-in transgenic sheep with reporter gene (tGFP) that mimics endogenous gene expression for the same targeted loci. The method is simple, and flexible in design. Furthermore, the efficiency of obtaining transgenic founders is 12.5%. We propose that CRISPR/Cas9 mediated knock-in will become a standard method for the generation of transgenic sheep lines.

## Results

### Confirmation of Rosa26 locus

**Figure 1:**
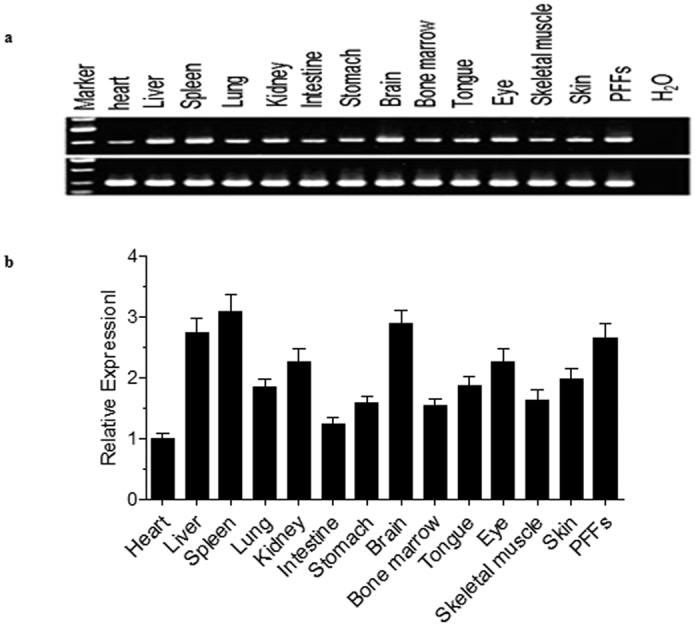
Characterization of *sRosa26* and highly efficient gene knock-in and replacement at the *sRosa26* locus. (**a**,**b**) *sRosa26* was expressed in a variety of organ tissues as determined by (**a**) RT-PCR and (**b**) quantitative RT-PCR. For RT-PCR, the designed primers annealed in *sRosa26 sequence* and amplified a correctly spliced product of 485 bp. Ovis *GAPDH* served as a control (234 bp). For qPCR, primers were specific to the *sRosa26* sequence. The PCR product of the Ovis *ACTB* gene served as the reference control. Data are presented as the average expression levels of three individual RT/qPCR reactions.

The ensemble ovis database was searched using the mouse Rosa26 sequence as a template and a highly conserved region was located on the sheep chromosome 19 (Supplementary Table S1). Sequence alignments of porcine, murine, rat, and human Rosa26 sequences showed high sequence conservation (>70%) among these species (Supplementary Fig. S1). According to the homologous sequence between them. In mice, rats, and humans, the Rosa26 locus encodes non-coding RNAs that are ubiquitously expressed. RT-PCR and SYBR-green-based quantitative PCR assays demonstrated that this non-coding RNA was expressed in a wide variety of adult sheep tissues (Fig. 1a,b).

### Designation of the sgRNA for sRosa26 locus

**Figure 2:**
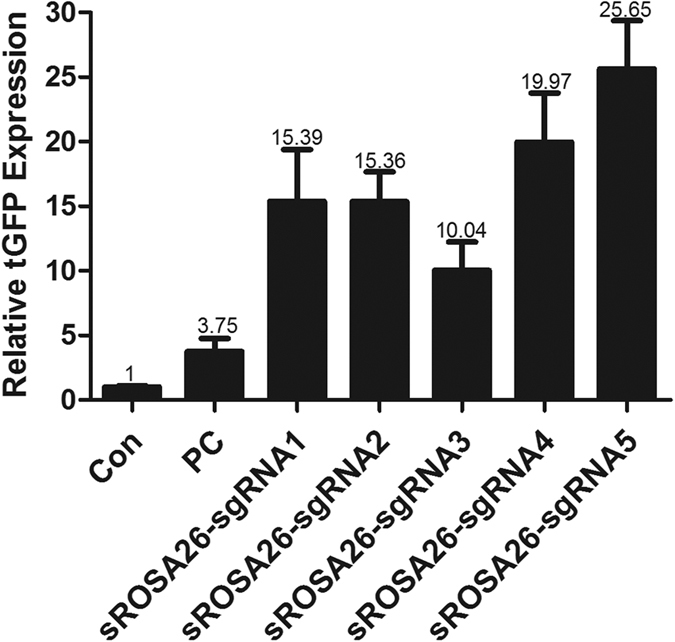
(**a**) Analysis of sgRNA activity. 5 guide RNAs (sgRNAs) were designed to target the sequence of the sRosa26 locus. All 5 sgRNAs efficiently guided Cas9 for genome editing.

Here, 5 guide RNAs (sgRNAs) were designed to target the sequence of the *sRosa26* locus (Fig. 2 and Table 1). The activity of the CRISPR/Cas9 was assessed using a UCA kit. The results of genotyping with the UCA kit showed that all 5 sgRNAs efficiently guided Cas9 for genome editing (Fig. 2a). This led to non-homologous end-joining mediated insertions and deletions (indels) in the *sRosa26* locus. The sgRNA with the highest activity was used to target the *sRosa26* locus.

### Preparation of the CRISPR/Cas plasmids for genome engineering

**Figure 3:**
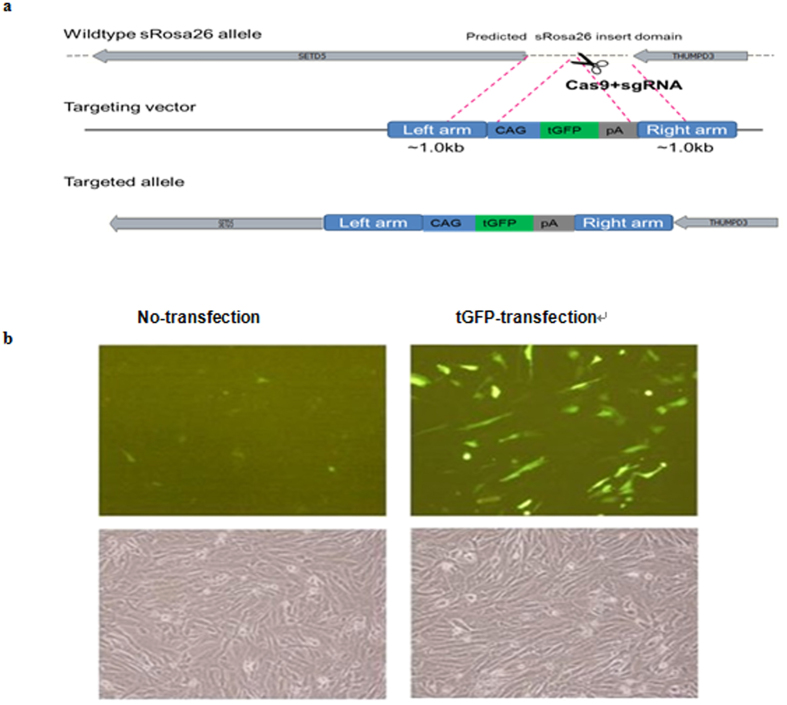
(**a**) Diagram of Cas9-mediated knock-in of LA-CAG-tGFP-polyA-RA into the sRosa26 locus. LA, left arm (1.0 kb); RA, right arm (1.0 kb). (**b**) *CAG* promoter-driven tGFP expression in sheep fibroblasts (*CAG* promoter region is shown in supplementary information).

The targeting vector contains a 1.0 Kb left arm and a 1.0 Kb right arm for homologous recombination (Fig. 3a). It also contains a CAG promoter (2753 bp, Supplementary Fig. S2). To validate the targeting efficiency of these sgRNAs, expression vectors were constructed in which a sgRNA was driven by the CAG promoter. An expression cassette comprising a viral splice acceptor (SA), a CAG promoter gene, and an *tGFP* gene was inserted between the homologous arms (Fig. 3a). The tGFP (928 bp, Supplementary Table S2) expression vector was designed to determine whether the exogenous gene can be expressed at the *sRosa26* locus. The CAG promoter-driven tGFP vector was transfected into sheep fibroblasts. At 48 h post-transfection, a high level of tGFP expression was detected in sheep fibroblasts (Fig. 3b).

### Cas9/sgRNA-Mediated Genome Targeting Efficiently Transmits into Ovaries of Sheep

**Figure 4:**
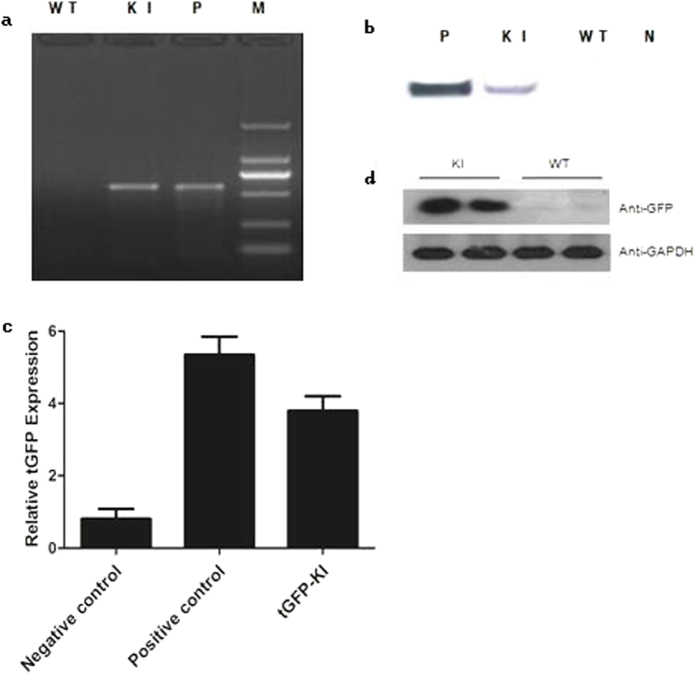
(**a**) PCR analysis of tGFP in tissues knock-in lambs. M = marker, N = negative control, P = postive control The size of target band is about 650 bp. (**b**) Southern blot analysis of tGFP–KI mutant sheep, Both the negative control and tGFP-KI sheep can be detected the target sequence. (**c**) RT- PCR analysis of tGFP expression in tissues knock-in lambs. All the lambs were 1 month old. tGFP gene expression quantity is 4.5 times that of the negative control. (**d**) Western blot analysis of tGFP–KI mutant sheep. Total protein from tGFP-KI sheep was subjected to SDS-PAGE on a 12% acrylamide gel. GADPH was used as a loading control. The tGFP gene expression cann’t be detected in controls.

The germline transmission of genetic mutations is the necessary condition to establish genetically modified animals^17^. *In vitro* synthesized Cas9 mRNA (40 ng/μl), sgRNA (20 ng/μl), and expression vector (20 ng/μl) were injected into the single-cell sheep embryos. Out of the 35 injected embryos, 30 healthy embryos were transferred into surrogate mothers and 8 pups were born (Fig. 4 and Table 2). Out of these 8 pups, 1 had the tGFP gene at the *sRosa26* locus and it was expressed in lamb ear tissues as indicated by PCR, Southern blot, RT-PCR and Western blot analysis (Fig. 4). And the sRosa26 locus sequence were detected by PCR and sequencing (Supplementary Fig. S3). The PCR and sequencing results showed that the tGFP gene locus was the sRosa26 target locus. These results suggest that the CRISPR/Cas9 system can induce mutations and knock-in DNA fragments in the *sRosa26* locus very efficiently.

## Discussion

Instead of knocking-in DNA constructs in the exon of a gene of interest, we knocked in constructs in the upstream region of a gene with the CAG promoter construct (Fig. 3a). There are advantages and a potential disadvantage in this approach. An advantage is that expression levels of transgenes are increased^18^. In our experience generating transgenic sheep, the usage of the CAG promoter instead of promoters of endogenous gene promoters increased the expression level of the transgene for many genes including tGFP. Another advantage is that a relatively large genomic region can be a subject of integration. This allowed that many sgRNAs can be tested by the usage of the CAG promoter. Transmission of the precise genome modification to the germline is a key to establishing a targeted gene knock-in line. In mice, rats, and humans, the Rosa26 locus encodes non-coding RNAs that are ubiquitously expressed^13^. So 5 guide RNAs (sgRNAs) were designed to target the sequence of the *sRosa26* locus.

In this study, we showed that the precise integration of exogenous DNA into the targeted genomic locus in sheep can be efficiently achieved using a donor vector containing short homology arms. We improved this method by introducing homology arms into a donor vector. In comparison to ZFNs and TALENs, the CRISPR/Cas9 system is suitable for this method because of the ease of donor-vector construction and of the multiple gRNA design^18^,^19^. A conventional knock-in vector carries more than 800 bp of homology arms without cleavable sites^20^,^21^. And the targeting vector contains a 1.0 Kb left arm and a 1.0 Kb right arm for homologous recombination.

The CRISPR/Cas9 system has been used to introduce defined genetic modification in cultured cell system^22^,^23^, but whether this could be applied to *in vivo* system and what parameter(s) is influential for its efficiency was less explored^24^. This is the first study to use oligonucleotides as templates to successfully knock DNA cassettes into the sheep genome via the CRISPR/Cas9 system. This is also the first recorded use of CRISPR/Cas9-mediated knock in of DNA sheep into the sRosa26 locus as part of a research exercise.

Collectively, these data strongly suggest that the *sRosa26* locus is an ideal site for expression of exogenous genes. But the using of Cas9-mediated knock-ins has not yet been reported in sheep. Here, this simple and efficient CRISPR/Cas9 system was successfully extended to the modification of the sheep genome at the *sRosa26* locus.

Unlike other ways of editing genes, which require construction of double-stranded DNA templates, this CRISPR/Cas9 system allows rapid and seamless editing of the genome at precise locations. It may become a powerful tool for assessing the functions of genes, altering critical residues in proteins to create desirable gain-of-function or loss-of-function mutations, and generating mutations in highly conserved proteins in sheep to facilitate the study of corresponding human diseases and agricultural production. All the results given above indicate that the sRosa26 locus is an excellent site for ubiquitous expression of exogenous genes.

Taken together, these results indicate that transgenic sheep stably overexpressing a gene of interest could be generated through CRISPR/Cas9 sysem at the sRosa26 locus without any need for more sophisticated technology.

## Methods

### Ethics statement

All animals were housed and handled according to China Agricultural University Institutional Animal Care and Use Committee guidelines and all animal work was approved by the appropriate committee (IACUC 0000000 and 0000000A-1). All experiments were performed according to institutional guidelines.

### Animals

All animals were handled according to the Guidelines for the Care and Use of Laboratory Animals established by the Beijing Association for Laboratory Animal Science. Animal experiments were approved by the Animal Ethics Committee of College of Biological Sciences of China Agricultural University.

### Cell culture

A sheep fetal fibroblast (SFB) cell line (China Agricultural University) was cultured in the medium containing 10% fetal bovine (Gibco, USA) serum in 5% CO_2_ at 37 °C. The cells were seeded in 6-well plates (Thermo Scientific, USA). After 24 h, cells were co-transfected with a mixture of plasmid, pcDNA3.1(+)-Cas9, pMD-19T-U6-sgRNA, and tGFP expression vector with the mass ratio of 2:1:1 (2500 ng in total per well), following the instruction of Lipofectamine3000 (Life Technologies, USA). Cells were harvested 48 h after transfection.

### Preparation of Cas9 mRNA and sgRNA

Cas9 and sgRNA coding regions containing the T7 promoter were PCR-amplified using DNA polymerase from each plasmid constructed above. The T7-Cas9 PCR products were gel purified and used as the template for *in vitro* transcription (IVT) using an mMESSAGE mMACHINE T7 ULTRA Transcription Kit (Life Technologies, USA). The T7-sgRNA PCR product was gel-purified and used as the template for IVT using MEGA shortscript T7 Kit (Life Technologies). The sgRNA was purified by ethanol precipitation, and all products were re-dissolved in RNase-free water and stored at −80 °C. 5 guide RNAs (sgRNAs) were designed to target the sequence of the *sRosa26* locus (Fig. 2 and Table 1). The activity of the CRISPR/Cas9 was assessed using a UCA kit (Beijing Biocytogen Co., Ltd, China).

### Microinjection of zygotes and embryo transfer

The sheep zygotes were obtained by super-ovulation of females and artificial insemination. The zygotes were flushed from the oviduct using sterile filtered embryo flushing solution. After that, 2–5 pl TE solution containing 20 ng/μl of sgRNA, 40 ng/μl of Cas9 mRNA and expression vector (20 ng/μl) were injected into the cytoplasm of pronucleus embryos using injection needles. Injections were performed using an Eppendorf TransferMan NK2 micromanipulator.

### Detection and Analysis of Indel Mutation in Lambs

Ear and muscle in hind leg were collected and digested in lysis buffer (10 μM Tris-HCl, 0.4 M EDTA, 1% SDS, and 100 μg/ml Proteinase K). And incubated at 50 °C for 1 hr, followed by extraction in 400 ml of phenolchloroform. The mixture was then centrifuged at 21,000 × g for 20 min at 4 °C. The supernatant was transferred to a new tube. An equal volume of isopropanol was added, and the tube was vortexed thoroughly. The mixture was then kept at −20 °C for at least 1 hr, followed by centrifugation at 21,000 × g for 20 min at 4 °C The supernatant was removed, and the DNA pellet was washed with 500 ml of 75% ethanol, Followed by centrifugation at 21,000 × g for 5 min at 4 °C. Finally, the pellet was dried for 10 min and resuspended in 30 ml of DNase-free water. For each samples, at least 100 clones were picked up randomly and sequenced.

### Southern Blot detection

To confirm transgene insertion in the sheep genome, Southern Blot was performed using DIG High Prime DNA Labeling and Detection Starter Kit II (Roche, 11585614910). DNA was isolate from the transgenic lambsand a wild type hu sheep tissue, Plasmid sRosa26-Gibson-HR1 was used as a positive control. The probe hybridizes to a 0.92 kb fragment depicted in Fig. 4b, indicating site-specific gene insertion.

### Western blotting analysis

For Western blot analysis, total proteins were isolated from the samples by homogenization in lysis buffer (50 mM Tris-HCl, pH 7.5, 150 mM NaCl, 1% Triton X-100, 0.25% sodium deoxycholate, and complete protease inhibitor cocktail (Roche). The concentration of proteins was measured by Bradford reagent (Sigma), separated on 10% SDS-PAGE gels and transferred to Immobilon-P membranes (Millipore). After blocking in 5% low-fat milk in PBST (0.1% Tween 20 in PBS) for 1 h, the membranes were incubated with tGFP antibody (1:500, SantaCruz Biotechnology), Fst antibody (1:500, Santa Cruz Biotech- nology) or mouse GAPDH antibody (1:2000, Santa Cruz Biotechnology) overnight at 4uC. After washing in PBST, the membranes were incubated in goat anti-rabbit antibody conjugated with horseradish peroxidase (1:5000) for 1 h, followed by three washes in PBST. The signals were detected by ECL Chemiluminescent kit (Amersham Pharmacia Biotech, Arlington Heights).

#### Off-target Analyses

Potential targets of tGFP gene and CAG promoter were detected using PCR amplifications with the program: 95 °C for 5 min; 95 °C for 1 min; 58 °C for 1 min, 72 °C for 1 min, 30 cycles, 72 °C for 10 min, store at 4 °C and the expression of tGFP gene were detected using RT-PCR, The target Rosa26 sequence were detected using PCR and Sanger sequencing (Invitrogen).

## Additional Information

**How to cite this article**: Wu, M. *et al*. *Rosa26*-targeted sheep gene knock-in via CRISPR-Cas9 system. *Sci. Rep*. **6**, 24360; doi: 10.1038/srep24360 (2016).

## Supplementary Material

Supplementary Information

## Figures and Tables

**Table 1:** 5 different sgRNA designations.

sgRNA		Sequence (5′–3′)	Target sequence length
sROSA26-sgRNA1	target sequence	ggaggcgatgacgagatcgc **ggg**	20 + PAM
	sROSA26-sgRNA1-Up	cacc ggaggcgatgacgagatcgc	
	sROSA26-sgRNA1-Dn	aaac gcgatctcgtcatcgcctcc	
sROSA26-sgRNA2	target sequence	ggggagggagggattcttct **agg**	20 + PAM
	sROSA26-sgRNA2-Up	cacc ggggagggagggattcttct	
	sROSA26-sgRNA2-Dn	aaac agaagaatccctccctcccc	
sROSA26-sgRNA3	target sequence	gacgagatcgcgggggaggg **agg**	20 + PAM
	sROSA26-sgRNA3-Up	cacc ggacgagatcgcgggggaggg	
	sROSA26-sgRNA3-Dn	aaac ccctcccccgcgatctcgtcc	
sROSA26-sgRNA4	target sequence	gaggcgatgacgagatcgcg **ggg**	20 + PAM
	sROSA26-sgRNA4-Up	cacc ggaggcgatgacgagatcgcg	
	sROSA26-sgRNA4-Dn	aaac cgcgatctcgtcatcgcctcc	
sROSA26-sgRNA5	target sequence	gtcgagtctctcctcgatta **tgg**	20 + PAM
	sROSA26-sgRNA5-Up	cacc ggtcgagtctctcctcgatta	
	sROSA26-sgRNA5-Dn	aaac taatcgaggagagactcgacc	

5 guide RNAs (sgRNAs) were designed to target the sequence of the *sRosa26* locus. The activity of the CRISPR/Cas9 was assessed using a UCA kit. All 5 sgRNAs efficiently guided Cas9 for genome editing.

**Table 2:** Numbers of injected and transferred embryos, newborns, and mutants generated during the establishment of knock-in sheep using gRNA/Cas9 system.

Gene	Number of embryos injected	Number of embryos transferred	Number of new borns	Number of mutations
sRosa26	35	30	8	1
Ration	/	85.7%	26.7%	12.5%

Cas9 mRNA and sgRNA were mixed and injected into sheep zygotes. The injected eggs were transferred into pseudopregnant females. The mutations were identified by sequencing PCR amplified 0.5 kbp genomic fragment containing target in the center. GMO; gene modified organism.
