# Supplementary material for: Rosa26-targeted sheep gene knock-in via CRISPR-Cas9 system
Source: Sci Rep. 2016 Apr 11;6:24360. doi: 10.1038/srep24360 (PMC4827023; doi:10.1038/srep24360)

Rosa26-targeted sheep gene knock-in via CRISPR-Cas9 system

Authors

Mingming Wu[,] ,Caihong Wei[,] ,Zhengxing Lian,Ruizao Liu[,] ,Caiye Zhu,Huihua Wang,Jiaxue Cao,Yuelei Shen,Fuping Zhao,Li Zhang,Zhu Mu,Yayu Wang, Xiaogang Wang,Lixin Du,Chuduan Wang.

Supplementary information

Table S1. Sheep Rosa26 sequence

GTCGTGAGGGTAGGTCTCTCTTTTCTGTTGGACCCTTACCTTGTCCCAGGCCTGGGCCCGGGCTGCGGCGCAC
GGCACTCCCGGGAGGCAGCAGGACTCGAGTTAGGCCCAACGCGGCGCCACGGCGTTTCCTGGCCGGGAATGG
CCCGTGCCCGTGAGGTGGGGGTGGGGGGCAGAAAGGCGGAGCGAGCCAAAGGCGGGGAGGGGGAAGGGCC
AGGGAAGGAGGGAGGGCCGGCACTACAGTGTTGGCGGACTGGCGGGACTGGGGCTGCGTGAGTCTCTGAGC
GCAGGCGGGCGGCGGCCGCCCCTCCCCCGGCGGCGGCGGCGGCGGCGGTAGCAGCTCACTCAGCCCGCTG
CCCGAGCGGAAACGCCACTGACCGCACGGGGATTCCCAGCGCCGGCGCCAGGGGCACCCGGGACACGCCCC
CTCCCGCCGCGCCATTGGCCCCTCCGCCCACCGCCCCGCACCCATTGGCCAACTCGCTGCCAATCAGCGGAA
GCCGCCGGGGCCACCTAGAGAAGAGGCTGTGCTCTGGGGCTCCGGCTCCTCAGAGAGCCTCGGCTAGGTAGG
GGAGCGGAACTCTGGTGGGAGGGGGTCCGGCGGATTGGTGGGGGGATGGGTGGCTGAGGTCGTCTGGCCGG
TATCTGGGGGTCGCCTTCCCCGGTGGGAAGACGGGAGCATAGCGTTTGTTACGCTGAAAGGGAAAGAGGTGGT
CAGAGGCAGGCGAGAGTGCGGCCCCCCTTGCGGCAACCGAAGGGGGAGGGAGAAGGGAGCGGAAAAGGCTC
GAATCCGGACGGAGCCATTGCTCCTGCAGAGGGAGGAGCGCTTCCGGCTCTTGTCTTGTCACTGATTGGTTGC
TGCTCCTCCCGCCGTGTGTGAAAACACAAATGGCGGGTTTTGGTTGGAGTAAAGCGCCTGTCAGTTACAGCCTC
GGGAGTGCGCAGCCGCCTAGGGACTCTCGCTTTGCCCACTGGGTGGGTGCTTAGGTAGGTAGGGTGGAGAGA
GACTTGGATGAGCAGGCGCGGTCGGCCTCCCACGGGGAGGTAGGGGTGGGGGTGGGAAGGGAGGGTCAGTG
AAAGTGGCTTCGCGCGGGCGTCCTACCACCCTCCCCTTCCTTCGGGGGAGTCGGTTTACCCGCCGCCTGCTTG
GCTTCGGCATCTGATTGGCTGCTGAAGCTCAGGGAACGGCCCCTTGTTATTGGCTCGGGTCCCAAATGAGCGA
AACCACTGCGCGGGTCGGCGGGGAGGCGGTCGTTGGTACGGTCCTCCCCGAGACCCAGCGCCCCAGTGTCTG
GCCCCGGGCCCCTGCGCAACGTGGCAGGAAGCGCGCGCTGGAGGTGGGGGCGGACTGCCGGGCCGAGGATT
CTGGGTGGTGGCGATTTCGGCTCCGCCCTGGGCGCCCGCTGCCTGAAGGACAAGACTAGCCCGACCTGCTCC
TGGACCCGTGGGGCTGAAGGGAGGAGTGGGGGTCGGTGCCGCTGGCTTGTGGGTGGGAGGTGCATGTTCTCC
AAAAATCGGCGCGAGCTGCAATCCTGAGGTGGCTGCAGTGGAGGAGGCGGAGAGAAGGCCGCACCCTTCTCA
GCAGGGGGAGGGGAGTGCCGCAATACCTTTATGGGAGTTCTCTGCTGCCTCCTGCCTCCTAAGGACCGCCCTG
GGCCTAGAAGAATCCCTCCCTCCCCCGCGATCTCGTCATCGCCTCCATGTCGAGTCTCTCCTCGATTATGGGCG

GGATTCTTTTGCCCAGGCTTAACCTTAATCCTGGGCGTTGTCCTGCAGTGGATCCAGCAGGTATAAGATTTAGAG

GACGAACCCAATTTCTTTATCTTCCCACAGACTTGAGTTTGTGTCACAAAATAATTATAATGGGGTGGTGGAGTG

AAATGAAGTCCAGGCAACACCTGGGACTGATTTTATGCAGCGAGACTGCGTGTTACTACTTAACATCCTTTTGTT

GCATTTTTTCCAGGAGATTGAGAGAGAGGTTAAAAGCTTGATCTCCTGAGTTTTATACTCTCCCCGTTTGAGACA

GTTGAGAAATAGGTTAAAGGCATGATCTCTTGAGTTCCCCATTTGAGACCGTTGCTACACCGCCAAAACAGAGC

GTCTTAGATTAGATCTTAAAAATTTAATTCCCCACCCTTGCATTCTCAGAGTCAGGCCTTTTAGCAACTCTCACTC

ATACTTTCAGCCCATTTTCTGTTTGTTATACTTGCTCAGCCTGTCCCAGTCATACCATTGGCTTTCTCCTCTCCTG

TTTTTGGTATCCCGGTGAGTCATGAAACTAGACAGGTTTCACCACCAATTAAGGCTACCTAGCTCGAGCATAGGC

TTCACTCTTGCCCAGCAATGCGTTTATTCCTCTTTTATGGATATTCTTGAGTCTTTACCTTGATATTCATTTAATTTT

TTAACCTCAGCTGGGATTCTACTGTCCCTCTTAGTAGTCCAGATGATCTTGACTACTGCTTTGCTGAGAACTGGA

CGTGAGGTTCAGCAGCATCTCTTTTATATCCTTAGAATACCTTTCAATCCATTTTCATTGATATGCTCATCAGTTAG

TAATCAAGCTCAGTTGCCATAAGGCTAGTATCCTCCGAACTAGTATCTCTTGTGTCTGGTATCTGCTGATACAACT

TTCATGTGTCCAGGACAGTTGTTCTCATACAAAGATACAGCATGGAAGTAACTGATCAACTCCTTTACTGGGTGG

TAACTACTGACAGGATGAATTCCATCATCACAAACGTGATGTG

Figure S1A Region conserved among mouse, rat, human ,pig and sheep Rosa26

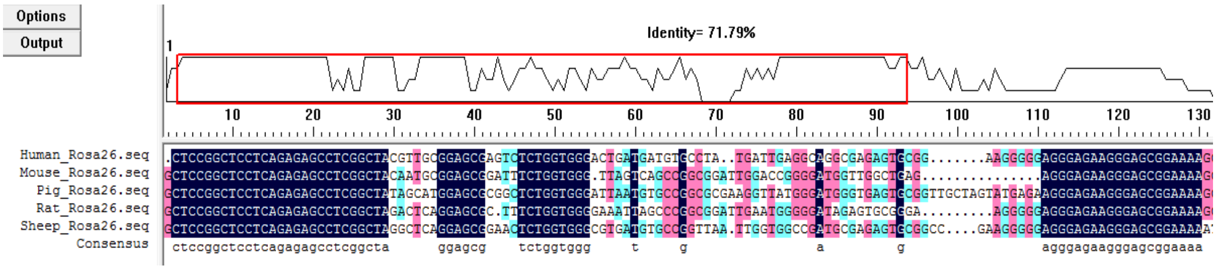

Figure S2 Targeting vector information.

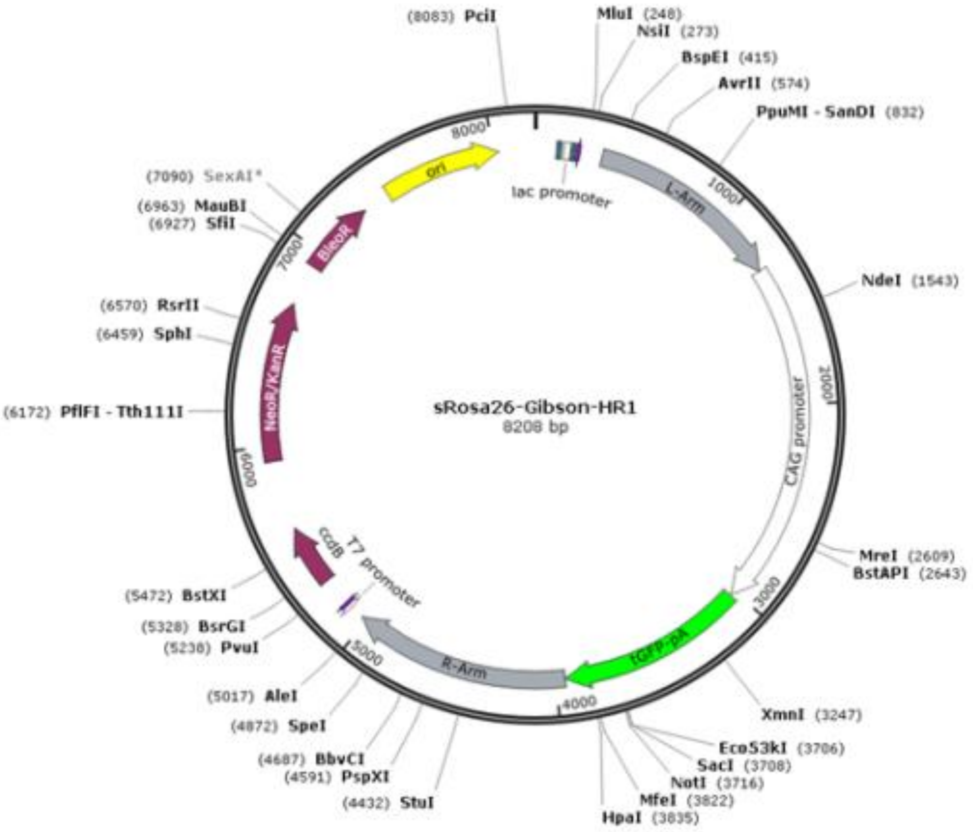

Table S2. tGFP sequence

CTAGTTATTAATAGTAATCAATTACGGGGTCATTAGTTCATAGCCCATATATGGAGTTCCGCGTTACATAACTTAC<br>GGTAAATGGCCCGCCTGGCTGACCGCCCAACGACCCCCGCCCATTGACGTCAATAATGACGTATGTTCCCATAG<br>TAACGCCAATAGGGACTTTCCATTGACGTCAATGGGTGGAGTATTTACGGTAAACTGCCCACTTGGCAGTACATC<br>AAGTGTATCATATGCCAAGTACGCCCCCTATTGACGTCAATGACGGTAAATGGCCCGCCTGGCATTATGCCCAG<br>TACATGACCTTATGGGACTTTCCTACTTGGCAGTACATCTACGTATTAGTCATCGCTATTACCATGGTCGAGGTG<br>AGCCCCACGTTCTGCTTCACTCTCCCCATCTCCCCCCCCTCCCCACCCCCAATTTTGTATTTATTTATTTTTTAAT<br>TATTTTGTGCAGCGATGGGGGCGGGGGGGGGGGGGGGGCGCGCGCCAGGCGGGGCGGGGCGGGGCGAGG<br>GGCGGGGCGGGGCGAGGCGGAGAGGTGCGGCGGCAGCCAATCAGAGCGGCGCGCTCCGAAAGTTTCCTTTT<br>ATGGCGAGGCGGCGGCGGCGGCGGCCCTATAAAAAGCGAAGCGCGCGGCGGGCGGGGAGTCGCTGCGACG<br>CTGCCTTCGCCCCGTGCCCCGCTCCGCCGCCGCCTCGCGCCGCCCGCCCCGGCTCTGACTGACCGCGTTACT<br>CCCACAGGTGAGCGGGCGGGACGGCCCTTCTCCTCCGGGCTGTAATTAGCGCTTGGTTTAATGACGGCTTGTT<br>TCTTTTCTGTGGCTGCGTGAAAGCCTTGAGGGGCTCCGGGAGGGCCCTTTGTGCGGGGGGAGCGGCTCGGGG<br>GGTGCGTGCGTGTGTGTGTGCGTGGGGAGCGCCGCGTGCGGCTCCGCGCTGCCCGGCGGCTGTGAGCGCTG<br>CGGGCGCGGCGCGGGGCTTTGTGCGCTCCGCAGTGTGCGCGAGGGGAGCGCGGCCGGGGGCGGTGCCCCG<br>CGGTGCGGGGGGGGCTGCGAGGGGAACAAAGGCTGCGTGCGGGGTGTGTGCGTGGGGGGGTGAGCAGGGG<br>GTGTGGGCGCGTCGGTCGGGCTGCAACCCCCCCTGCACCCCCCTCCCCGAGTTGCTGAGCACGGCCCGGCTT<br>CGGGTGCGGGGCTCCGTACGGGGCGTGGCGCGGGGCTCGCCGTGCCGGGCGGGGGGTGGCGGCAGGTGGG<br>GGTGCCGGGCGGGGCGGGGCCGCCTCGGGCCGGGGAGGGCTCGGGGGAGGGGCGCGGCGGCCCCCGGAG<br>CGCCGGCGGCTGTCGAGGCGCGGCGAGCCGCAGCCATTGCCTTTTATGGTAATCGTGCGAGAGGGCGCAGGG<br>ACTTCCTTTGTCCCAAATCTGTGCGGAGCCGAAATCTGGGAGGCGCCGCCGCACCCCCTCTAGCGGGCGCGG<br>GGCGAAGCGGTGCGGCGCCGGCAGGAAGGAAATGGGCGGGGAGGGCCTTCGTGCGTCGCCGCGCCGCCGTC<br>CCCTTCTCCCTCTCCAGCCTCGGGGCTGTCCGCGGGGGGACGGCTGCCTTCGGGGGGGACGGGGCAGGGCG<br>GGGTTCGGCTTCTGGCGTGTGACCGGCGGCTCTAGAGCCTCTGCTAACCATGTTCATGCCTTCTTCTTTTTCCTA<br>CAGCTCCTGGGCAACGTGCTGGTTATTGTGCTGTCTCATCATTTTGGCAAAGAATTACAACC

Figure S3. The PCR result of the tGFP gene knock in sRosa26 locus

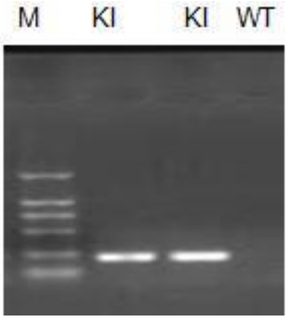

Figure S4.The sRosa26 locus sequencing of tGFP-KI sheep.

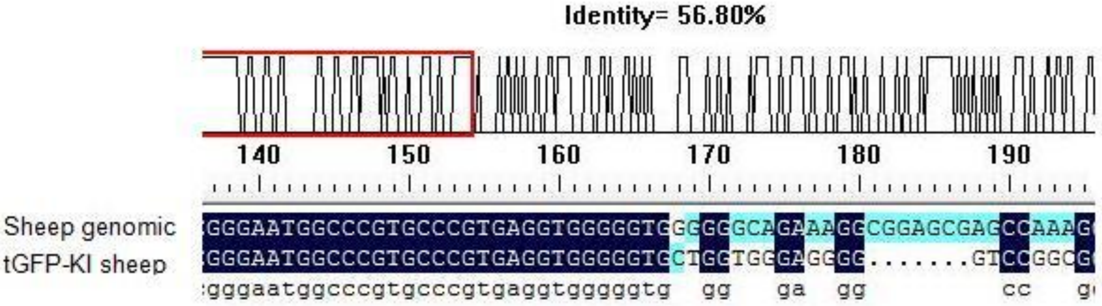

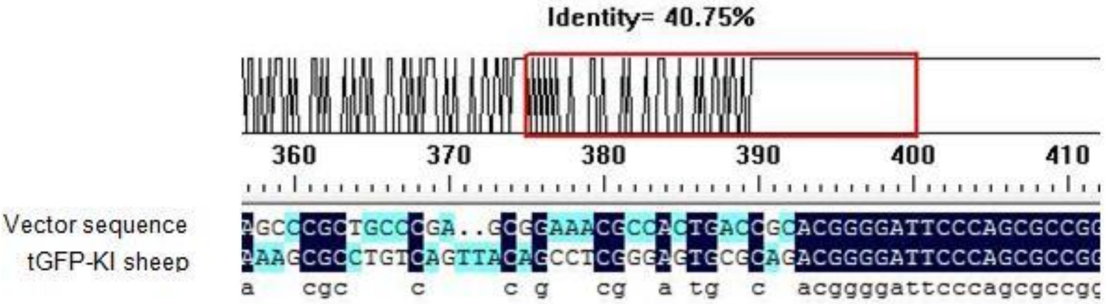

Supplement: Supplementary Information [file srep24360-s1.pdf]
